# Preoperative Assessment of Circulating Extracellular Vesicles as a Potential Biomarker for Melanoma

**DOI:** 10.3390/life16101705

**Published:** 2026-10-12

**Authors:** Giulio Gualdi, Paolo Amerio, Andrea Marani, Edvige Morea, Davide Bertolla, Patrick Silvetti, Davide Brocco, Nicola Tinari, Alessandro Cama, Pasquale Simeone, Giulia Catitti, Ignazio Stanganelli, Paola Lanuti

**Affiliations:** 1 University Hospital, Ospedale Maggiore di Parma, 43126 Parma, Italy; 2 Clinic of Dermatology, Department of Medicine and Aging Sciences, University G. d’Annunzio, 66100 Chieti, Italyvivimorea@gmail.com (E.M.); 3 Dermatologic Unit, Department of Surgery, AUSL Romagna, 47923 Rimini, Italy; 4 Section of Dermatology and Infectious Diseases, Department of Medical Sciences, University of Ferrara, 44121 Ferrara, Italy; 5 Department of Medical, Oral and Biotechnological Sciences, “G.d’Annunzio” University of Chieti-Pescara, 66100 Chieti, Italy; 6 Department of Pharmacy, “G.d’Annunzio” University of Chieti-Pescara, 66100 Chieti, Italy; 7 Center for Advanced Studies and Technology (CAST), “G.d’Annunzio” University of Chieti-Pescara, 66100 Chieti, Italy; pasquale.simeone@unich.it (P.S.);; 8 Dermatology Resident Training Program, Department of Medicine and Surgery, University of Parma, 43121 Parma, Italy; 9 Skin Cancer Unit, IRCCS Istituto Romagnolo per lo Studio dei Tumori “Dino Amadori”-IRST Meldola (FC), 47014 Meldola, Italy

**Keywords:** extracellular vesicles, cutaneous melanoma, liquid biopsy, PD-L1, VISTA, flow cytometry, biomarker

## Abstract

Background: Circulating extracellular vesicle (EV)-associated particle profiles may differ between patients with melanoma and those with benign lesions. Methods: Preoperative citrated whole blood from 77 patients with clinically suspicious lesions was assessed by direct fluorescence-based flow cytometry; histopathology identified 20 CMM cases (12 primary and 8 metastatic) and 57 benign-lesion controls. Results: The reported total particle concentration was higher in CMM than controls (3299.86 ± 456.61 vs. 2873.40 ± 427.74 events/µL). CD45+, PD-L1+, and CD45+/PD-L1+ populations were also increased. CD45+/VISTA+ particles were higher in the reduced subset of 9 CMM cases and 48 controls. Conclusions: These exploratory observations require analytical validation and independent clinical confirmation before diagnostic or prognostic utility can be established.

## 1. Introduction

Extracellular vesicles (EVs) are nanosized membrane-bound particles released by virtually all cell types that selectively transport proteins, lipids, nucleic acids, and other cytoplasmic and membrane components [1]. By mediating intercellular communication, EVs regulate a wide range of physiological and pathological processes and are increasingly recognized as promising diagnostic biomarkers and therapeutic targets [2]. Although EVs have traditionally been classified as microvesicles, exosomes, and apoptotic bodies, the considerable overlap in their size, molecular composition, and biogenesis has prompted the International Society for Extracellular Vesicles to recommend a simplified classification based primarily on particle size, distinguishing small EVs (<200 nm) from medium/large EVs (>200 nm) [3]. In oncology, EVs have emerged as key regulators of tumour initiation, progression, metastatic dissemination, and therapeutic resistance, highlighting their potential as valuable biomarkers and therapeutic targets [4,5].

Cutaneous malignant melanoma (CMM) is an aggressive malignancy arising from melanocytes and is characterized by remarkable clinical, dermoscopic, histopathological, and molecular heterogeneity [6,7,8,9,10]. Although its incidence is substantially lower than that of other skin cancers, CMM accounts for the vast majority of skin cancer-related deaths because of its high metastatic potential and its frequent occurrence in relatively young individuals, representing a major clinical and socioeconomic burden [11]. Tumour thickness, measured as Breslow thickness, remains the most important histopathological prognostic factor, whereas increasing evidence indicates that both mitotic rate and diagnostic delay are also closely associated with patient prognosis [12,13,14].

Growing evidence indicates that EVs actively contribute to CMM progression by promoting tumour growth, immune evasion, metastatic spread, and remodelling of the tumour microenvironment [15]. Patients with CMM exhibit higher circulating EV concentrations than healthy individuals in several published series [14,16,17,18]. In addition, melanoma-derived EVs mediate bidirectional communication between tumour cells and immune cells, exerting both immunostimulatory and immunosuppressive effects depending on their molecular cargo [19,20]. Recent studies have also investigated circulating EV profiles in relation to immune checkpoint inhibitor response and other immunotherapeutic strategies [19,21,22,23]. More broadly, EV-mediated intercellular communication, metastatic signalling and therapeutic resistance have also been described in other solid tumours, including breast cancer, underscoring the wider oncological relevance of EV biology [24].

However, most available evidence has been generated using melanoma cell lines, experimental animal models, or blood samples collected from patients with an already established diagnosis of CMM, frequently after surgical excision and/or systemic treatment. Consequently, little is known about the circulating EV profile present before any therapeutic intervention in patients undergoing evaluation for skin lesions clinically suspected of CMM. Liquid biopsy encompasses several analytically distinct circulating biomarkers, including EVs and cell-free DNA; recent work on long cell-free DNA illustrates the rapid development of complementary liquid-biopsy approaches while also emphasizing that analytical validation is required before clinical implementation [25].

In the present study, peripheral blood samples were collected before surgical excision of skin lesions clinically suspected of CMM. Following histopathological diagnosis, circulating EV-associated events were quantitatively and phenotypically characterized and compared between patients with histologically confirmed CMM and subjects whose lesions were histopathologically negative for CMM, thereby establishing a clinically relevant control group. Particular attention was paid to selected immunophenotypic subpopulations to explore whether their distributions differed between groups; the study was not designed or powered to establish diagnostic or prognostic performance.

## 2. Materials and Methods

### 2.1. Study Population

This prospective observational study enrolled consecutive patients referred to the Dermatology Units of the “SS. Annunziata” Hospital (Chieti, Italy) and the “G. Bernabeo” Hospital (Ortona, Italy) between January and October 2021 for the surgical excision of skin lesions clinically suspected of cutaneous malignant melanoma (CMM).

Peripheral blood samples were collected immediately before surgery, prior to histopathological diagnosis. Following histopathological examination, patients with histologically confirmed primary CMM, with or without metastatic disease, were included in the study group. Subjects whose excised lesions were histopathologically negative for CMM and showed no evidence of malignancy constituted the control group.

The exclusion criteria were age <18 years or >75 years, previous or concomitant malignancies, autoimmune or chronic inflammatory diseases, and ongoing immunosuppressive therapy.

### 2.2. Ethics Statement

The study was conducted in accordance with the principles of the Declaration of Helsinki and was approved by the Ethics Committee of G. d’Annunzio University, Chieti, Italy (Ethics Approval Code: CE-2016-0147; Protocol No. 3271/16; approval date: 25 February 2016). Written informed consent was obtained from all participants prior to enrollment and blood sample collection. A blank copy of the informed consent form, in both the original language (Italian) and its English translation, has been provided as Supplementary Material.

### 2.3. Flow Cytometric Characterization of Extracellular Vesicles

Peripheral blood was collected into sodium citrate tubes (BD Biosciences; original manuscript reference 454387) before surgery and processed within 4 h. Five microlitres of unseparated whole blood were incubated with the antibody panels for 45 min at room temperature in the dark, then diluted with 500 µL of 1× PBS; 1 × 10^6^ events per sample were acquired on a FACSVerse flow cytometer. No plasma preparation or physical EV isolation step was reported. Accordingly, the assay measures fluorescence-defined circulating particle events directly in whole blood; it does not establish that every event represents an isolated EV. The contribution of platelets, erythrocytes, lipoproteins and non-vesicular particles was not separately quantified.

The acquisition threshold was set on the APC fluorescence channel, as in the previously validated LCD-based EV workflow. Height signals were displayed on biexponential or logarithmic scales. Instrument performance was monitored using the BD Cytometer Setup & Tracking Module; compensation used CompBeads and single-stained controls, and FMO controls defined marker positivity. Data were analysed using FACSuite v1.0.6.5230 and FlowJo X v10.0.7, and concentrations were obtained by volumetric counting. The Rosetta Calibration approach used by the group was applied for dimensional standardization of EV-associated events, following the methodology reported by Simeone et al. and Marchisio et al.

### 2.4. Statistical Analysis

Statistical analyses were designed to compare circulating extracellular vesicle (EV) concentrations and immunophenotypic profiles between patients with cutaneous malignant melanoma (CMM) and non-CMM controls, as well as to investigate associations between EV concentrations and clinicopathological characteristics.

Continuous variables were summarized as mean ± standard deviation (SD) or median and interquartile range (IQR), according to their distribution. Categorical variables were expressed as absolute frequencies and percentages. The normality of continuous variables was assessed using the Shapiro–Wilk test and visual inspection of distribution plots.

Comparisons of continuous variables between patients with CMM and non-CMM controls were performed using the independent-samples Student’s *t*-test for normally distributed data or the Mann–Whitney U test for non-normally distributed data. For comparisons among patients with primary CMM, metastatic CMM, and non-CMM controls, one-way analysis of variance (ANOVA) or the Kruskal–Wallis test was used, as appropriate. When an overall group difference was identified, post hoc pairwise comparisons were performed using Tukey’s test or Dunn’s test, respectively.

Categorical variables were compared using the chi-square test or Fisher’s exact test, as appropriate.

The analysis of CD45+/VISTA+ EV-associated events was restricted to the available subgroup of 9 of 20 CMM cases and 48 of 57 non-CMM controls. Thus, VISTA measurements were unavailable for 11/20 CMM cases (55.0%) and 9/57 controls (15.8%).

All statistical tests were two-sided. A nominal *p*-value < 0.05 was used for exploratory comparisons. No adjustment for multiple comparisons was performed; therefore, all reported *p*-values, including the *p* = 0.047 comparison for total particle concentration, are explicitly interpreted as nominal and exploratory.

### 2.5. EV Characterization and Analytical Validation

Circulating extracellular vesicles (EVs) were characterized using a fluorescence-based flow cytometric approach, following previously established methodological procedures. The analysis was performed directly on peripheral whole-blood samples, allowing the simultaneous quantification and immunophenotypic characterization of circulating EV-associated particles without an additional EV isolation step.

EV-associated events were identified using the lipophilic cationic dye (LCD) and the antibody panels detailed in Table 1. Phalloidin staining was included in the analytical panel, while CD45, CD90, CD117, CD146, PD-L1, and VISTA were included as phenotypic markers. CD146 was included in the acquisition panel; however, no quantitative CD146 outcome is reported in the analytical dataset available for this manuscript. CD146 was therefore not included in the comparative outcome analyses or biological interpretation, and no conclusion regarding CD146 can be drawn from this study (Table 2).

Flow cytometric acquisition was performed using a FACSVerse flow cytometer (BD Biosciences, San Jose, CA, USA). The acquisition threshold was set on the allophycocyanin (APC) fluorescence channel, and all parameters were acquired and analyzed using height signals displayed on biexponential or logarithmic scales.

Fluorescence Minus One (FMO) controls were employed to define marker positivity and distinguish positive events from background fluorescence. Fluorescence compensation was performed using single-stained controls and CompBeads (BD Biosciences). Instrument performance and fluorescence standardization were monitored using the Cytometer Setup & Tracking Module (BD Biosciences).

**Figure 1:**
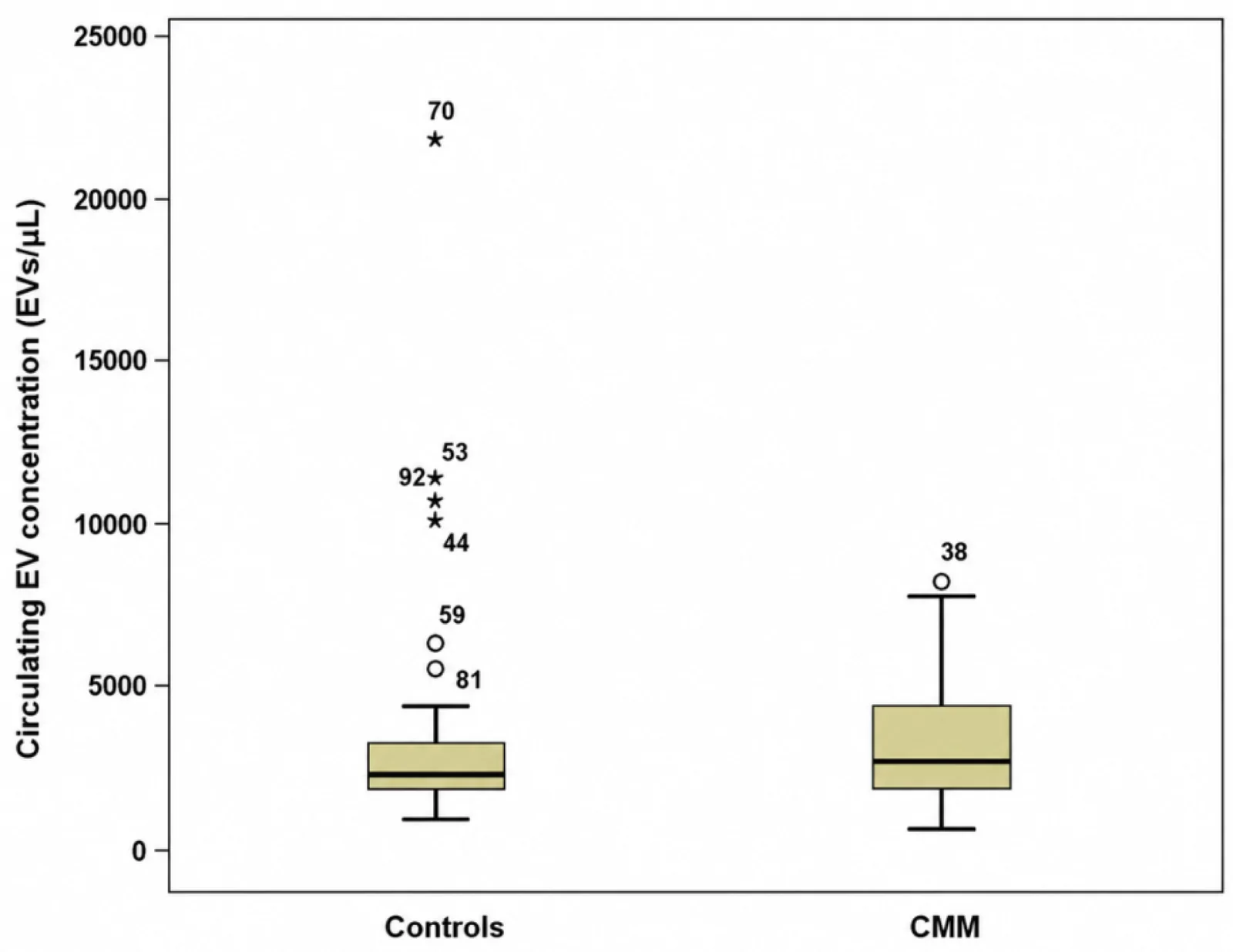
Study boxplots of total circulating fluorescence-defined particle concentration in benign-lesion controls and CMM.

The gating strategy applied in this cohort followed the previously validated whole-blood flow-cytometric workflow described by Simeone et al. and Marchisio et al. Briefly, acquisition was triggered on the APC channel in which the lipophilic cationic dye (LCD) emits. A platelet-free region was first defined using scatter/size information; events within this region were then displayed on a Phalloidin FITC-H/LCD-H plot, and LCD-positive/Phalloidin-negative events were selected as the EV-enriched population. Phenotypic gates were subsequently applied within this population for CD90, CD117, CD45, PD-L1 and VISTA, with marker positivity defined using the corresponding fluorescence-minus-one controls. The resulting populations included CD90+, CD117+, CD90+/CD117+, CD45+, PD-L1+, CD45+/PD-L1+ and CD45+/VISTA+ events. The methodological gating sequence is summarized in Figure 1.

Flow cytometric data were analyzed using FACSuite software (version 1.0.6.5230, BD Biosciences) and FlowJo X (version 10.0.7, BD Biosciences). Absolute concentrations of circulating EV-associated events were determined by volumetric counting and expressed as EVs/µL.

The analytical approach was based on a previously published flow cytometric methodology for circulating EV characterization. However, independent confirmation of EV identity and size distribution by Western blotting, nanoparticle tracking analysis (NTA), dynamic light scattering (DLS), or transmission electron microscopy (TEM) was not documented for the present cohort. Consequently, the detected populations were characterized according to their fluorescence and immunophenotypic profiles, without assigning a specific EV biogenesis pathway or establishing the vesicular nature of every detected event.

### 2.6. Calibration and Analyte Estimation

Absolute concentrations of circulating EV-associated events were estimated by volumetric flow cytometry and expressed as events/µL of blood. Particle enumeration was based on the number of events meeting the predefined fluorescence and immunophenotypic gating criteria within the analyzed sample volume. Accordingly, the reported concentrations represent flow cytometry-derived estimates of detectable EV-associated events rather than concentrations obtained from an analyte-specific standard curve.

Routine instrument performance was monitored using the Cytometer Setup & Tracking procedure, and fluorescence compensation was performed using single-stained controls and compensation beads.

## 3. Results

### 3.1. Study Population and Baseline Characteristics

A total of 98 consecutive patients were enrolled in the study. Twenty-one patients were excluded because of blood sample processing issues, resulting in a final study population of 77 subjects. Following histopathological examination, 20 patients were diagnosed with cutaneous malignant melanoma (CMM), whereas 57 subjects with lesions histopathologically negative for CMM constituted the control group. The CMM cohort comprised 12 patients with primary CMM and 8 with metastatic CMM, as shown in Table 3

**Table 3:** Available baseline characteristics.

Characteristic	CMM (n = 20)	Benign-Lesion Controls (n = 57)
Age, years, mean ± SD	61.4 ± 13.2	55.8 ± 15.1
Female, n (%)	9 (45.0%)	31 (54.4%)
Male, n (%)	11 (55.0%)	26 (45.6%)
Primary CMM, n (%)	12 (60.0%)	—
Metastatic CMM, n (%)	8 (40.0%)	—

Study flow (reported): 98 enrolled; 21 excluded due to processing issues; 77 analyzed (20 CMM and 57 controls).

### 3.2. Total Circulating Particle Concentration

**Figure 2:**
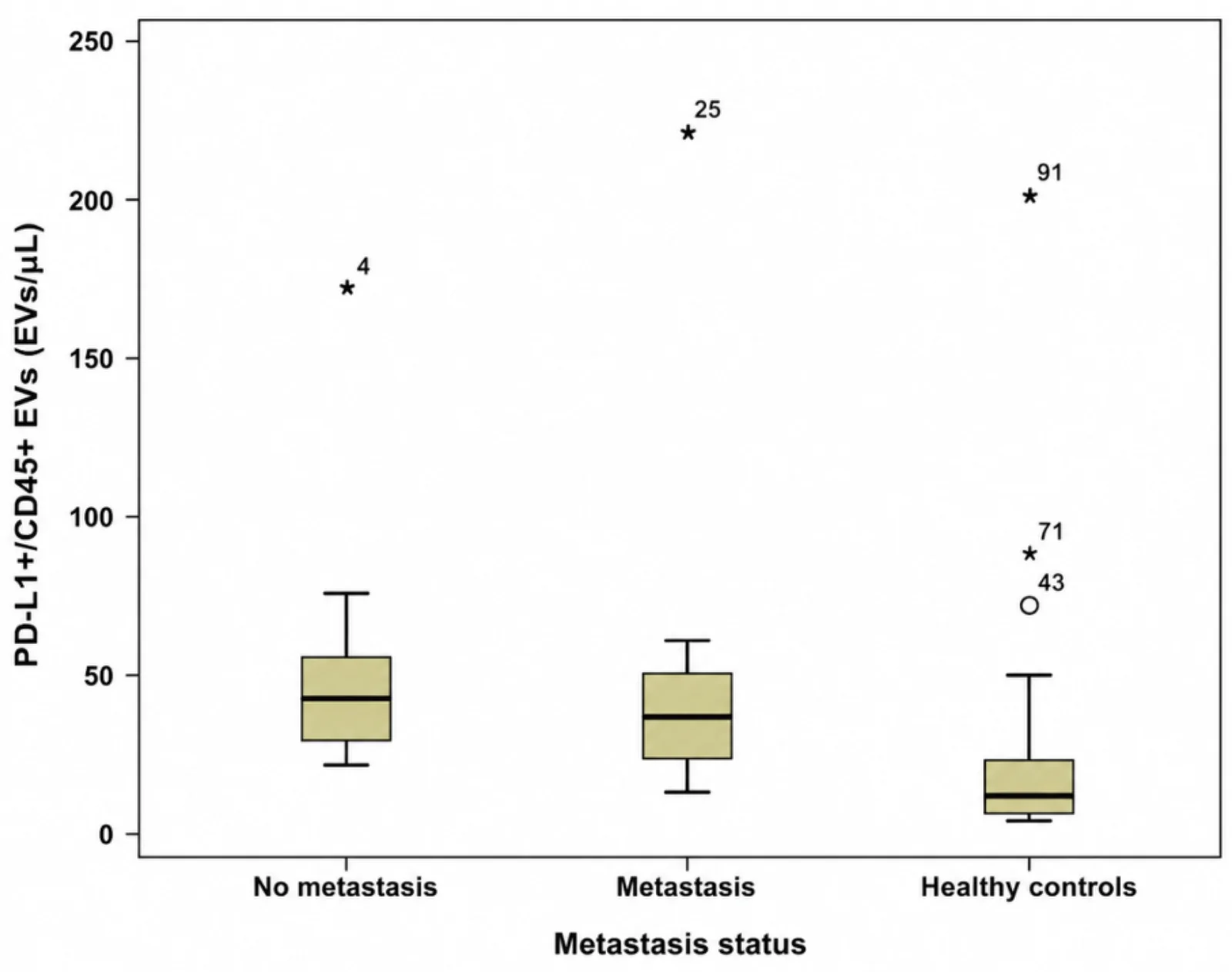
Study boxplots of CD45+/PD-L1+ particle concentrations by primary CMM (no metastasis), metastatic CMM and benign-lesion controls.

The reported total circulating fluorescence-defined particle concentration was higher in CMM than controls (3299.86 ± 456.61 vs. 2873.40 ± 427.74 events/µL; nominal *p* = 0.047; Figure 2).

The reported phenotypes included CD90+, CD117+, CD90+/CD117+, CD45+, PD-L1+, CD45+/PD-L1+ and CD45+/VISTA+ populations. CD45 positivity is consistent with hematopoietic association but does not establish a particular leukocyte lineage; PD-L1 positivity alone does not establish tumor origin.

### 3.3. Immune-Related Particle Populations

Reported CD45+, PD-L1+ and CD45+/PD-L1+ concentrations are shown in Table 4.

**Figure 3:**
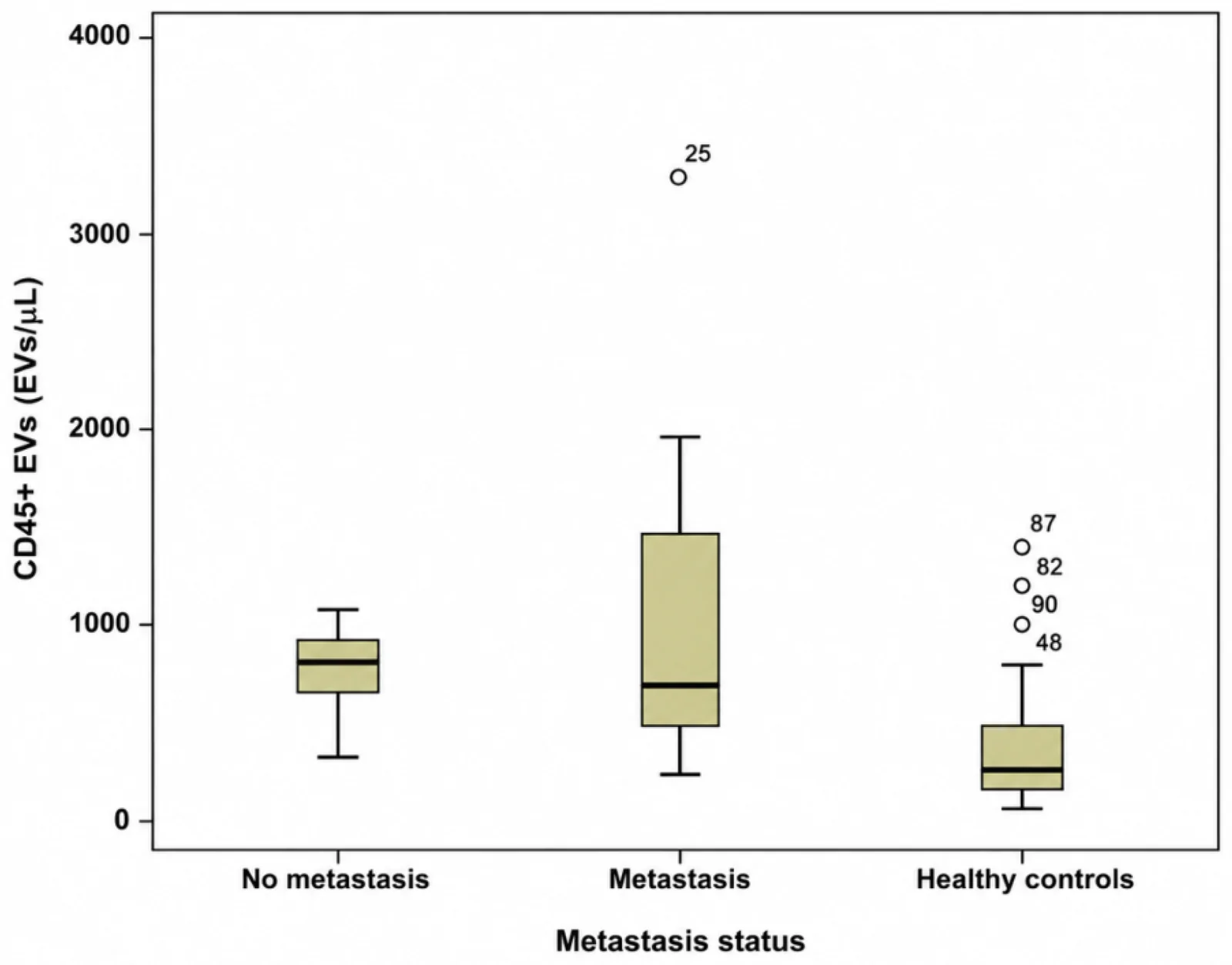
Study boxplots of CD45+/PD-L1+ particle concentrations by primary CMM (no metastasis), metastatic CMM and benign-lesion controls.

No statistically significant differences between primary and metastatic CMM were reported for the analysed particle populations (Figure 3).

### 3.4. CD45+/VISTA+ Subset Analysis

**Figure 4:**
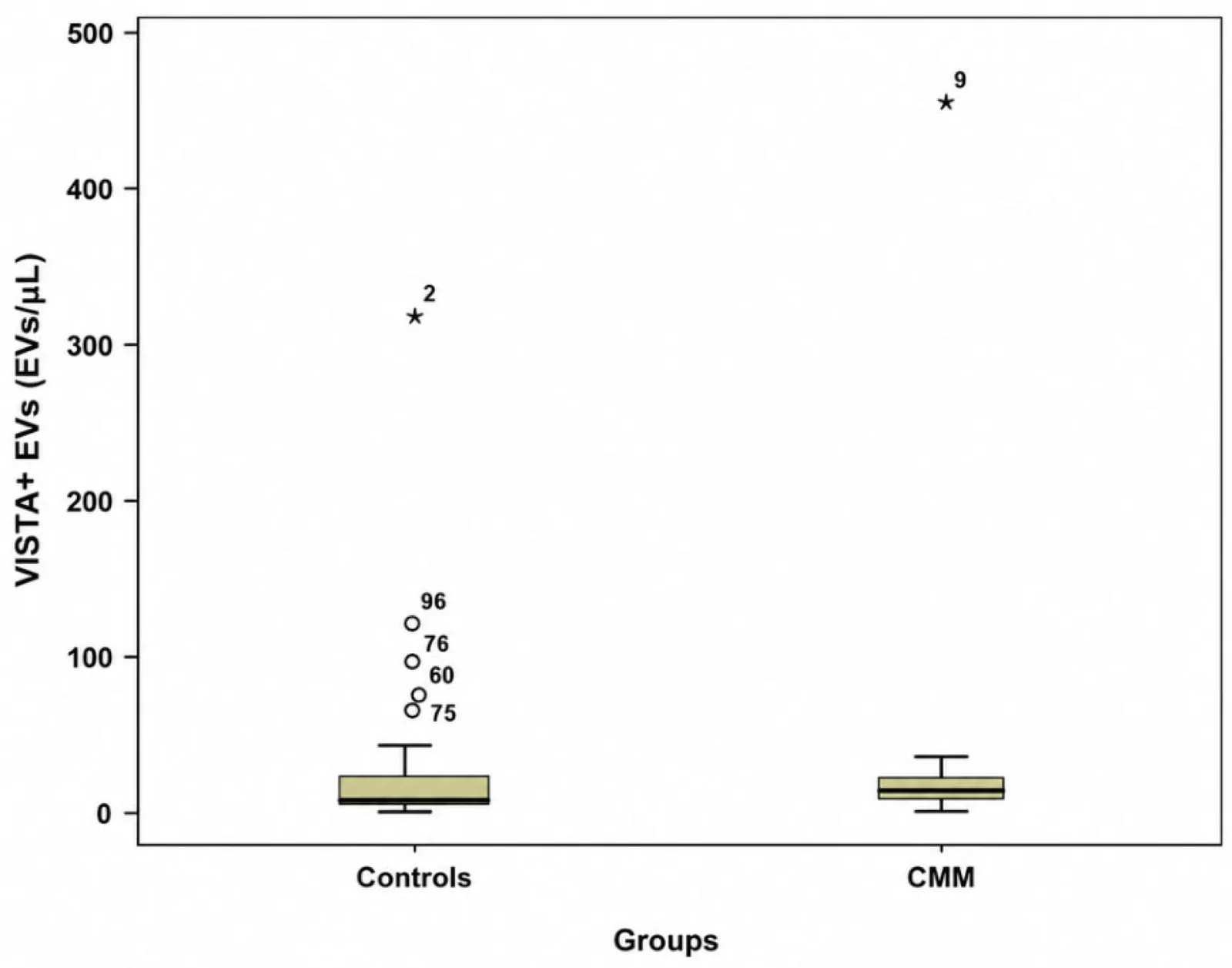
Study boxplots of CD45+ particle concentrations by primary CMM (no metastasis), metastatic CMM and benign-lesion controls.

**Figure 5:**
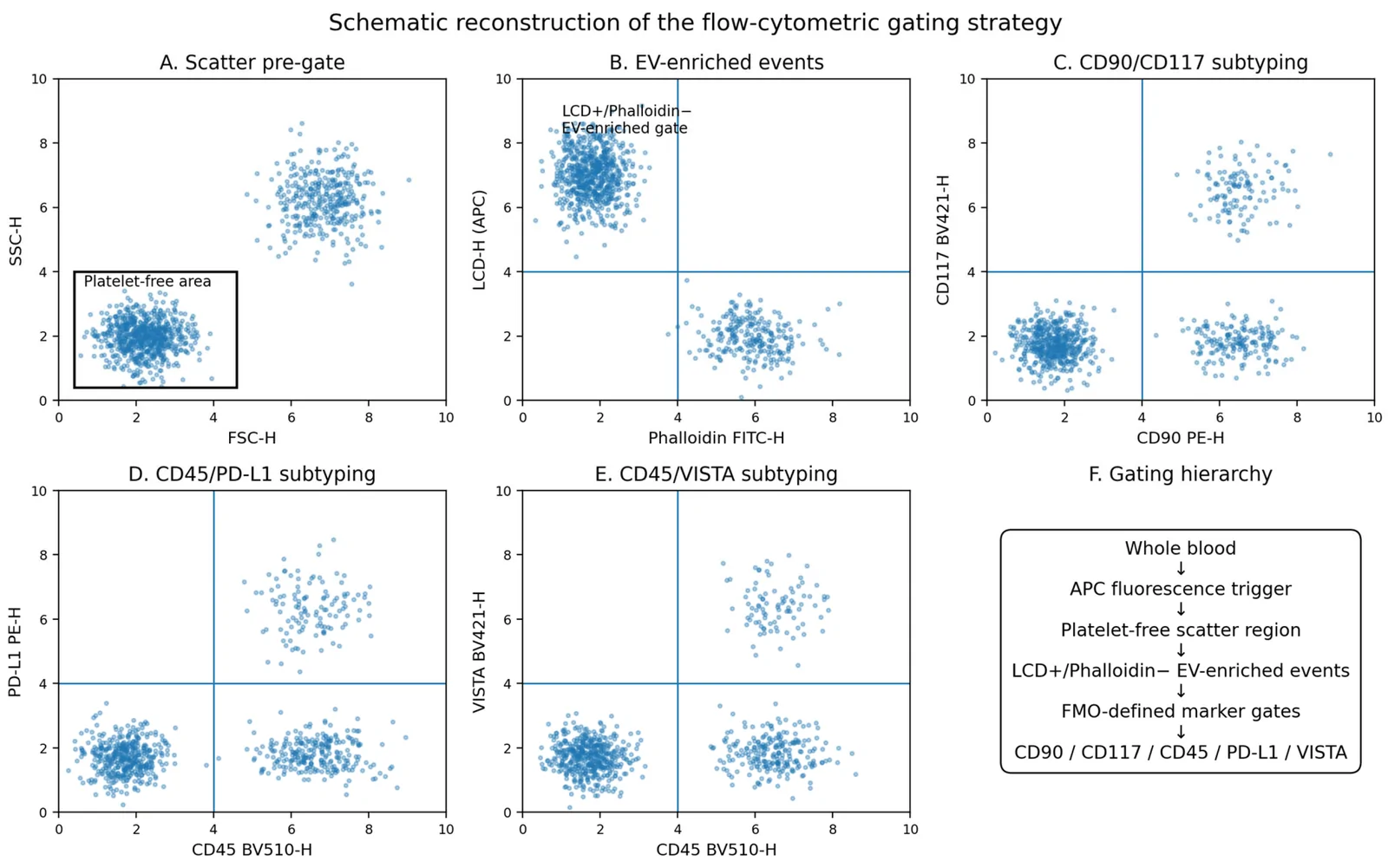
Study boxplots of CD45+/VISTA+ particle concentrations in CMM (n = 9) and benign-lesion controls (n = 48).

CD45+/VISTA+ EV-associated events were available for 9 of 20 CMM cases and 48 of 57 non-CMM controls (65.678 ± 49.5473 vs. 20.321 ± 6.7312 events/µL; nominal *p* = 0.031; Figure 4). CD45+/VISTA+ EV-associated events were evaluated in a subset of 9 patients with cutaneous malignant melanoma (CMM) and 48 non-CMM controls. The reported mean concentration was higher in patients with CMM than in controls (65.678 ± 49.5473 vs. 20.321 ± 6.7312 events/µL, respectively; nominal *p* = 0.031; Figure 5).

This analysis was restricted to the subset for which CD45+/VISTA+ measurements were reported. The result should therefore be interpreted as an exploratory finding rather than evidence of a validated association in the full study population.

## 4. Discussion

The present study explored circulating EV-associated particle profiles in cutaneous malignant melanoma (CMM) using peripheral blood collected before surgical excision and histopathological diagnosis. The data show group-level differences in total fluorescence-defined particle concentration and in selected immune-related phenotypes.

The involvement of EVs in cancer biology has attracted considerable attention over the last decade. Increasing evidence suggests that EVs actively participate in tumour progression by mediating intercellular communication, modulating the tumour microenvironment, promoting metastatic dissemination, and contributing to immune escape and therapeutic resistance [26,27,28,29]. Accordingly, circulating EVs have been proposed as potential biomarkers in several malignant and non-malignant diseases, including cardiometabolic, neurological, infectious, and neoplastic disorders [26,27,28]. Our findings are consistent with this growing body of evidence and further support the hypothesis that circulating EVs may reflect biological processes associated with CMM.

One aspect of the present study is its preoperative sampling design. Unlike investigations based on samples obtained after an established melanoma diagnosis or treatment, peripheral blood in this study was collected before surgical excision and before histopathological confirmation of CMM. The control group consisted of subjects undergoing excision of clinically suspicious lesions that were subsequently shown to be benign. This provides a clinically relevant sampling context for exploratory comparison of circulating EV-associated profiles.

To investigate the cellular origin of circulating EVs, we analysed different phenotypic subsets. CD90 and CD117 were selected as markers potentially associated with tumour-derived EVs. CD90 has been reported to be expressed by circulating melanoma cells and by specific melanoma variants, including spindle cell melanoma [30]. Similarly, CD117 (c-KIT), a receptor tyrosine kinase involved in cell proliferation and differentiation, has been implicated in the biology of several malignancies, including melanoma, prostate cancer, and gastrointestinal stromal tumours [31,32,33]. In addition, proteomic analyses have identified KIT among the proteins enriched in melanoma-derived exosomes, suggesting its possible involvement in metastatic progression [34].

The CD90+, CD117+ and CD90+/CD117+ group values are retained as originally reported. The CD90+/CD117+ value for metastatic CMM was numerically lower than the control value.

The observed differences in CD45+, PD-L1+ and CD45+/PD-L1+ particle populations may reflect a mixture of cellular sources and pre-analytical influences.

PD-L1 represents one of the best-characterised immune checkpoint molecules in contemporary oncology (Figure 1). Tumour PD-L1 expression is used as a predictive biomarker in several malignancies, although its clinical utility in CMM remains context-dependent. Previous studies have demonstrated PD-L1 on melanoma-derived exosomes and have investigated circulating PD-L1-positive EVs in relation to immunotherapy response [35,36,37,38]. More broadly, current cancer-immunotherapy research continues to investigate additional immune checkpoints and signalling pathways beyond PD-1/PD-L1, including TIM-3, LAG-3, TIGIT and intracellular signalling networks [39,40]. The present observation of higher PD-L1+ and CD45+/PD-L1+ particle concentrations is therefore interpreted only as an exploratory immunophenotypic difference (Figure 2)

The VISTA result requires particularly cautious interpretation. Measurements were available for only 9/20 melanoma cases and 48/57 controls, corresponding to missing VISTA data in 55.0% and 15.8% of the two groups, respectively

## 5. Conclusions

Circulating extracellular vesicle (EV)-associated events exhibited differences in concentration and immunophenotypic profile between patients with cutaneous malignant melanoma and non-CMM controls. These findings suggest that flow cytometric assessment of circulating EV-associated populations may provide information relevant to melanoma research. Further studies using standardized EV characterization methods, larger patient cohorts, and independently validated analytical approaches are required to establish the clinical significance of these findings.

## Figures and Tables

**Table 1:** Flow cytometry antibody panels used for the phenotypic characterization of circulating extracellular vesicles (EVs).

Panel	Target/Reagent	Fluorochrome	Manufacturer	Clone	Catalog No.	Volume/Test (µL)
1	Lipophilic Cationic Dye (LCD)	-	BD Biosciences	-	626267	0.5
1	Phalloidin	FITC	BD Biosciences	-	626269	0.5
1	CD90	PE	BD Biosciences	OX-7	551401	3
1	CD146	PE-Cy7	BD Biosciences	P1H12	562135	3
1	CD117	BV421	BD Biosciences	YB5.B8	562434	3
1	CD45	BV510	BD Biosciences	HI30	626287	5
2	LCD	-	BD Biosciences	-	626267	0.5
2	Phalloidin	FITC	BD Biosciences	-	626269	0.5
2	PD-L1	PE	BD Biosciences	MIH1	557924	1
2	VISTA	BV421	BD Biosciences	MIH65	566750	0.2
2	CD45	BV510	BD Biosciences	HI30	626287	5

**Table 2:** Quantification of circulating CD90+, CD117+, and CD90+/CD117+ extracellular vesicle (EV) subpopulations in patients with cutaneous malignant melanoma (CMM) and controls.

EV Subset (EVs/µL, Mean ± SD)	Primary CMM	Metastatic CMM	Controls
CD90+ EVs	92.320 ± 17.5882	121.188 ± 62.3719	71.767 ± 19.0610
CD117+ EVs	178.550 ± 32.1162	255.716 ± 120.1932	181.384 ± 70.5067
CD90+/CD117+ EVs	20.730 ± 6.8692	9.908 ± 4.2464	18.703 ± 8.3159

**Table 4:** Quantification of circulating immune-related extracellular vesicle (EV) subpopulations in patients with cutaneous malignant melanoma (CMM) and controls.

EV Subset (EVs/µL, Mean ± SD)	Primary CMM	Metastatic CMM	Controls	*p* Value
Total CD45+ EVs	683.7093 ± 66.4	1112.8253 ± 422.60720	280.1827 ± 34.55607	0.001
Total PD-L1+ EVs	272.4497 ± 52.8973	212.9235 ± 53.1763	200.8745 ± 45.11	0.004
CD45+/PD-L1+ EVs	52.026 ± 15.8485	55.923 ± 26.6556	18.625 ± 4.2290	0.001

## Data Availability

The aggregated data supporting the findings of this study are included within the article. The original individual-level datasets are not publicly available due to privacy and ethical considerations related to human participant data.

## Supplementary Materials

The following supporting information can be downloaded at: https://www.mdpi.com/article/10.3390/life16101705/s1.
