# Supplementary material for: Preoperative Assessment of Circulating Extracellular Vesicles as a Potential Biomarker for Melanoma
Source: Life (Basel). 2026 Oct 12;16(10):1705. doi: 10.3390/life16101705 (PMC13706239; doi:10.3390/life16101705)
Supplement: Supplementary file 1 [file life-16-01705-s001.zip › life-4537039-supplementary.pdf]

INFORMATIVA AL PAZIENTE E MODULO DI CONSENSO INFORMATO

Studio osservazionale sulle vescicole extracellulari circolanti nei pazienti con lesioni cutanee sospette per melanoma

Gentile Signora/Egregio Signore,
Le proponiamo di partecipare a uno studio osservazionale condotto presso le Unità di Dermatologia coinvolte. Prima di decidere se partecipare, La invitiamo a leggere attentamente questa informativa e a chiedere al medico qualsiasi chiarimento ritenga necessario. La partecipazione è libera e volontaria e l’eventuale rifiuto o ritiro del consenso non avrà conseguenze sul Suo percorso assistenziale.

1. Scopo dello studio

Le vescicole extracellulari (EVs) sono piccole strutture rilasciate dalle cellule e presenti anche nel sangue. Lo studio intende valutare la quantità e alcune caratteristiche delle EVs circolanti in pazienti sottoposti ad asportazione di lesioni cutanee clinicamente sospette per melanoma. I risultati delle analisi saranno valutati alla luce dell’esame istopatologico della lesione e, ove pertinente, dei principali dati clinico-patologici.

2. Perché Le viene proposta la partecipazione

Le viene proposta la partecipazione perché è prevista l’asportazione chirurgica di una lesione cutanea che necessita di esame istopatologico per escludere o confermare una diagnosi di melanoma. La partecipazione allo studio non modifica l’indicazione né le modalità dell’intervento chirurgico.

3. Procedure previste

In caso di adesione, prima dell’intervento chirurgico e prima che sia disponibile la diagnosi istopatologica verrà effettuato un prelievo di sangue periferico. Sul campione saranno eseguite analisi di ricerca mediante citometria a flusso per la quantificazione e caratterizzazione fenotipica delle vescicole extracellulari circolanti. Non sono previste procedure chirurgiche aggiuntive rispetto a quelle indicate per motivi clinici.

La lesione cutanea verrà sottoposta a prelievo bioptico e/o escissione chirurgica secondo l’indicazione clinica già stabilita nell’ambito del normale percorso diagnostico-assistenziale. Il tessuto prelevato sarà sottoposto a esame istopatologico per la definizione diagnostica. I risultati istopatologici e i principali parametri della lesione potranno essere correlati, in forma codificata, con i risultati delle analisi sulle vescicole extracellulari. La partecipazione allo studio non comporta l’esecuzione di una biopsia aggiuntiva esclusivamente a fini di ricerca, salvo quanto espressamente previsto dal protocollo approvato.

4. Possibili rischi e disagi

I rischi sono quelli associati a un normale prelievo venoso e possono comprendere dolore o fastidio transitorio, piccolo ematoma, lieve sanguinamento e, raramente, reazione vasovagale o lipotimia.

Per il prelievo bioptico/escissione della lesione si applicano i rischi propri della procedura clinicamente indicata, tra cui dolore locale, sanguinamento, ematoma, infezione, ritardo di cicatrizzazione ed esito cicatriziale. Tali rischi sono connessi alla procedura diagnostico-terapeutica e non specificamente alla partecipazione allo studio.

5. Possibili benefici

Non è previsto un beneficio clinico diretto derivante dalla partecipazione. Le analisi sulle EVs hanno finalità di ricerca e non sostituiscono gli accertamenti diagnostici della pratica clinica. I risultati potranno contribuire alle conoscenze sui biomarcatori circolanti nel melanoma.

6. Partecipazione volontaria e ritiro

La partecipazione è volontaria. Lei potrà rifiutare o ritirare il consenso in qualsiasi momento, senza dover fornire motivazioni e senza conseguenze sulla qualità dell'assistenza o sul rapporto con i curanti.

7. Riservatezza e trattamento dei dati

I dati personali, clinici e relativi ai campioni saranno trattati nel rispetto della normativa applicabile e, per le finalità di ricerca, saranno utilizzati in forma codificata/pseudonimizzata ove previsto. I risultati potranno essere presentati o pubblicati in forma tale da non consentire l'identificazione diretta dei partecipanti.

8. Utilizzo dei campioni biologici

Il campione di sangue sarà utilizzato per le analisi previste dal progetto di ricerca. L'eventuale conservazione di materiale residuo o il suo utilizzo per ulteriori finalità dovrà avvenire esclusivamente se previsto dal protocollo, dalle autorizzazioni applicabili e dal consenso del partecipante.

9. Costi e compensi

La partecipazione non comporta costi aggiuntivi e non prevede compensi economici.

10. Approvazione etica

Lo studio è condotto secondo i principi etici applicabili alla ricerca biomedica e della Dichiarazione di Helsinki. I riferimenti del Comitato Etico e dell'approvazione devono essere riportati esclusivamente sulla base della documentazione ufficiale.

Data di approvazione 25 Febbraio 2016

Responsabile dello studio Professor. Amerio Paolo

Contatto paolo.amerio@unich.it

DICHIARAZIONE DI CONSENSO INFORMATO

Nome e cognome del partecipante

____

Data di nascita ____

Dichiaro di aver ricevuto informazioni chiare e comprensibili sullo studio, di aver avuto tempo sufficiente per valutarne la partecipazione e la possibilità di porre domande, e di aver ricevuto risposte soddisfacenti.

- Ho compreso lo scopo e le procedure previste dallo studio.
- Ho compreso i possibili rischi e disagi legati al prelievo di sangue.
- Ho compreso che la partecipazione è volontaria e che posso ritirare il consenso in qualsiasi momento senza conseguenze sulla mia assistenza.
- Sono stato/a informato/a sulle modalità di tutela e trattamento dei miei dati.
- Ho compreso che i risultati della ricerca potranno essere pubblicati o presentati senza consentire la mia identificazione.
- Ho compreso che le analisi sperimentali non sostituiscono gli esami diagnostici della normale pratica clinica.

ACCONSENTO LIBERAMENTE E VOLONTARIAMENTE ALLA PARTECIPAZIONE ALLO STUDIO

Firma del partecipante ____

Luogo e data ____

CONSENSO AL PRELIEVO E ALL’UTILIZZO DEL CAMPIONE BIOLOGICO

Acconsento al prelievo del campione di sangue e al suo utilizzo per le analisi descritte nella presente informativa, nei limiti del protocollo e delle autorizzazioni applicabili.

Firma del partecipante ____

Luogo e data ____

DICHIARAZIONE DEL MEDICO/RICERCATORE

Dichiaro di aver fornito al/alla partecipante informazioni comprensibili sullo studio, sulle procedure, sui possibili rischi e benefici e sulla natura volontaria della partecipazione, e di aver risposto alle domande poste.

Nome e qualifica ____

Firma ____

Luogo e data ____

PATIENT INFORMATION SHEET AND INFORMED CONSENT FORM

Observational study of circulating extracellular vesicles in patients with skin lesions clinically suspected of melanoma

Dear Sir/Madam,
You are being invited to participate in an observational study conducted at the participating Dermatology Units. Before deciding whether to participate, please read this information carefully and ask the study physician any questions you may have. Participation is entirely voluntary. Refusal to participate or subsequent withdrawal of consent will not affect your medical care.

1. Purpose of the study

Extracellular vesicles (EVs) are small structures released by cells and found in blood. This study aims to evaluate the concentration and selected characteristics of circulating EVs in patients undergoing surgical excision of skin lesions clinically suspected of melanoma. Research findings will be evaluated in relation to the histopathological diagnosis and, where applicable, relevant clinical and pathological data.

2. Why you are being invited

You are being invited because you are scheduled to undergo surgical excision of a skin lesion requiring histopathological examination to exclude or confirm melanoma. Participation will not alter the indication for or conduct of your surgical procedure.

3. Study procedures

If you agree to participate, a peripheral venous blood sample will be collected before surgery and before the histopathological diagnosis is available. The sample will undergo research analyses using flow cytometry to quantify and phenotypically characterize circulating extracellular vesicles. No additional surgical procedures are required for study participation.

The skin lesion will undergo biopsy and/or surgical excision according to the clinical indication already established as part of routine diagnostic care. The tissue specimen will undergo histopathological examination for diagnostic purposes. Histopathological findings and relevant lesion parameters may be correlated, in coded form, with the results of extracellular-vesicle analyses. Participation in this study does not require an additional biopsy solely for research purposes, unless this is specifically provided for in the approved study protocol.

4. Possible risks and discomforts

The risks are those associated with routine venous blood collection and may include temporary pain or discomfort, minor bruising, mild bleeding and, rarely, a vasovagal reaction or fainting.

Biopsy and/or excision of the skin lesion carries the risks associated with the clinically indicated procedure, including local pain, bleeding, bruising, infection, delayed wound healing and scarring. These risks are related to the diagnostic/therapeutic procedure itself and not specifically to study participation.

5. Possible benefits

No direct clinical benefit is expected. EV analyses are performed for research purposes and do not replace routine diagnostic examinations. The results may contribute to knowledge concerning circulating biomarkers in melanoma.

6. Voluntary participation and withdrawal

Participation is voluntary. You may decline or withdraw your consent at any time without giving a reason and without any effect on the quality of your medical care or your relationship with your treating physicians.

7. Confidentiality and data processing

Personal, clinical and sample-related data will be processed in accordance with applicable data-protection requirements and, for research purposes, will be coded/pseudonymized where appropriate. Study findings may be presented or published in a manner that does not permit direct identification of individual participants.

8. Use of biological samples

The blood sample will be used for the analyses specified in the research project. Any storage of residual material or use for additional research purposes must occur only if permitted by the study protocol, applicable approvals and the participant's consent.

9. Costs and compensation

Participation involves no additional costs and no financial compensation is provided.

10. Ethical approval

The study is conducted in accordance with applicable ethical principles for biomedical research and the Declaration of Helsinki. Ethics Committee and approval details must be entered only on the basis of official documentation.

Approval date 25 February 2016

Principal Investigator Professor Amerio Paolo

Contact paolo.amerio@unich.it

INFORMED CONSENT STATEMENT

Participant full name ____

Date of birth ____

I confirm that I have received clear and understandable information about the study, have had sufficient time to consider participation, have had the opportunity to ask questions, and have received satisfactory answers.

- I understand the purpose of the study and the procedures involved.
- I understand the possible risks and discomforts associated with blood collection.
- I understand that participation is voluntary and that I may withdraw consent at any time without affecting my medical care.
- I have been informed about the protection and processing of my data.
- I understand that research findings may be published or presented without permitting my identification.
- I understand that experimental analyses do not replace routine diagnostic examinations.

I FREELY AND VOLUNTARILY CONSENT TO PARTICIPATE IN THIS STUDY

Participant signature ____

Place and date ____

CONSENT TO BLOOD COLLECTION AND USE OF BIOLOGICAL SAMPLE

I consent to the collection of a blood sample and its use for the analyses described in this information sheet, within the limits of the study protocol and applicable approvals.

Participant signature ____

Place and date ____

INVESTIGATOR/PHYSICIAN STATEMENT

I confirm that I have provided the participant with understandable information about the study, its procedures, potential risks and benefits, and the voluntary nature of participation, and that I have answered the participant’s questions.

Name and professional role ____

Signature ____

Place and date ____
